# Sequence signature analysis of chromosome identity in three *Drosophila *species

**DOI:** 10.1186/1471-2105-6-158

**Published:** 2005-06-23

**Authors:** Per Stenberg, Fredrik Pettersson, Anja O Saura, Anders Berglund, Jan Larsson

**Affiliations:** 1 UCMP, Umeå University, Umeå, Sweden; 2 Research Group for Chemometrics, Department of Chemistry, Umeå University, Umeå, Sweden; 3 Department of Genetics, University of Helsinki, Helsinki, Finland

## Abstract

**Background:**

All eukaryotic organisms need to distinguish each of their chromosomes. A few protein complexes have been described that recognise entire, specific chromosomes, for instance dosage compensation complexes and the recently discovered autosome-specific Painting of Fourth (POF) protein in *Drosophila*. However, no sequences have been found that are chromosome-specific and distributed over the entire length of the respective chromosome. Here, we present a new, unbiased, exhaustive computational method that was used to probe three *Drosophila *genomes for chromosome-specific sequences.

**Results:**

By combining genome annotations and cytological data with multivariate statistics related to three *Drosophila *genomes we found sequence signatures that distinguish Muller's F-elements (chromosome 4 in *D. melanogaster*) from all other chromosomes in *Drosophila *that are not attributable to differences in nucleotide composition, simple sequence repeats or repeated elements. Based on these signatures we identified complex motifs that are strongly overrepresented in the F-elements and found indications that the *D. melanogaster *motif may be involved in POF-binding to the F-element. In addition, the X-chromosomes of *D. melanogaster *and *D. yakuba *can be distinguished from the other chromosomes, albeit to a lesser extent. Surprisingly, the conservation of the F-element sequence signatures extends not only between species separated by approximately 55 Myr, but also linearly along the sequenced part of the F-elements.

**Conclusion:**

Our results suggest that chromosome-distinguishing features are not exclusive to the sex chromosomes, but are also present on at least one autosome (the F-element) in *Drosophila*.

## Background

Eukaryotes need to distinguish their individual chromosomes in several essential processes. For example, homologous chromosomes must be aligned during meiosis and the nuclear positioning of specific chromosomes in interphase is conserved, controlled and important for correct gene expression [1,2]. Therefore, there must be certain molecules, most likely proteins, that recognise chromosome-specific features. However, few protein complexes that recognise a specific chromosome and bind at multiple points along its entire length are known. One example is the MSL ribonucleoprotein complex of *Drosophila melanogaster*, a dosage compensation factor that equalises expression of sex-linked genes between males and females – an essential process in animals with an XX-XY mode of sex determination [3-5]. In this species an autosome-specific protein has also been discovered; the Painting of fourth (POF) protein, which binds exclusively to the 4^th ^chromosome [6]. The chromosome specificity of POF and MSL appears to have been conserved for a long period of evolutionary history, suggesting that chromosome-specific identifying features have also been conserved and have functional significance in *Drosophila *[7]. The fact that individual chromosomes can be uniquely targeted raises questions about how they are recognised. There are no known DNA targets that direct the binding of either the MSL complex or POF, and no sequences have previously been found that are chromosome-specific and distributed over its entire length.

The development of appropriate computational methods is essential for linking functions to linear sequences. The toolbox for finding cryptic and complex sequence targets is still developing and most algorithms require extensive optimisation or a known motif [for a review of current methods see [8]]. Standard methods, e.g. BLAST and alignment approaches used in these kinds of analyses have several limitations, for instance they are neither exhaustive nor unbiased. To circumvent these limitations, Brāzma et al. [9] successfully combined a pattern discovery algorithm with clustering of expression data to predict gene regulatory elements in yeast [see also [10]]. We present here an alternative way to analyse large amounts of sequence data using multivariate statistics, combined with cytological observations and full genome annotations, to find sequence signatures composed of combinations of sequence motifs correlated to chromosomal regions without imposing any predefined assumptions. The multivariate approach is efficient in finding weak signals in large amounts of data. The method is neither biased nor heuristic, but still very fast. Abe et al. [11] used a related approach to study large-scale differences between distant genomes, and Bultrini et al. [12] used sequence motifs and multivariate statistics to find vocabularies defining intron regions in *Drosophila melanogaster *and *Caenorhabditis elegans*. In the study reported here, our aim was to use a multivariate approach to identify sequence signatures correlated to chromosome identity in *Drosophila*, and if possible link these signatures to function. The *D. melanogaster *genome sequence (release 3) [13] and its annotation (release 3.2) [14] have been thoroughly revised since their first releases and the cytology of the salivary gland polytene chromosomes (X, 2, 3, 4) provides a powerful tool for genome studies. Although the *D. yakuba *(2004-04-07 assembly) and *D. pseudoobscura *(Freeze 1) genome assemblies are not as complete as that of the *D. melanogaster *assembly, they provide valuable resources for attempts to identify conserved, potentially functional sequences. The three *Drosophila *species examined in this study (*D. yakuba*, *D. pseudoobscura *and *D. melanogaster*) all belong to the *Sophophora *subgenus and are hereafter referred to as *Dy*, *Dp *and *Dm*, respectively. *Dm *and *Dy *both belong to the *melanogaster *species group and *Dp *to the *obscura *species group. *Dm *and *Dy *are separated by approximately 12.8 Myr and they are both separated from *Dp *by roughly 54.9 Myr [15].

## Results

### Whole chromosome analysis

**Figure 1:**
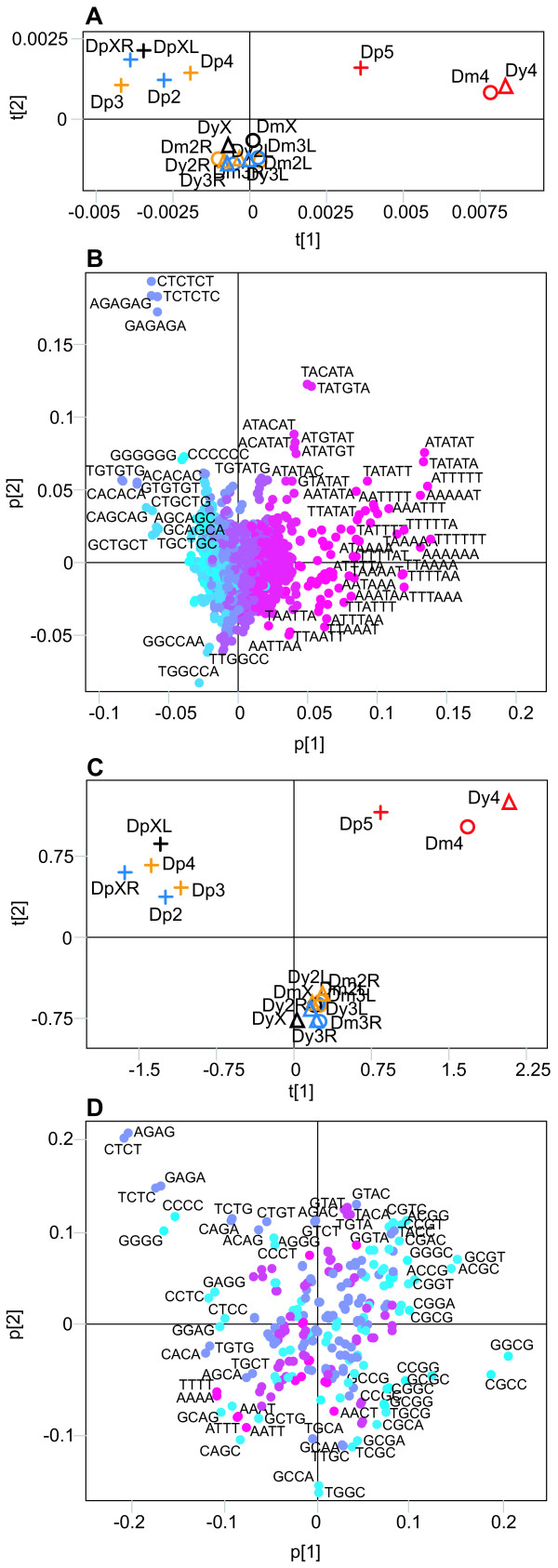
Results of the PCA of whole chromosome sequences from *Dm *(○), *Dy *(△) and *Dp *(+). Chromosomes are colour-coded, as follows (according to the *Dm *numbering: black = X, yellow = 2, blue = 3 and red = 4). L and R stand for the left and right arms of the metacentric chromosomes, respectively. (**A**) Score plot (R2 = 0.87) of the non-normalised hexamer analysis. (**B**) Loading plot of the analysis in (**A**). (**C**) Score plot (R2 = 0.84) of the normalised tetramer analysis. (**D**) Loading plot of the analysis in (**C**). The colouring of the hexamers in (**B**) and (**D**) is proportional to the A/T content. Pink is all A/T and blue is all G/C.

To construct data sets for a whole genome analysis, we scored all positions of all possible di-(16), tri-(64), tetra-(256), penta-(1024) and hexa-mers (4096) in the genome sequence of *Dm*, *Dy *and *Dp*. PCA (Principal Component Analysis) of the scores clearly separated the Muller's F-elements (the term F-element is used here because this chromosome is the 4^th ^in *Dm*/*Dy *and the 5^th ^in *Dp *[16]) from all other chromosomes along the first component (Figure 1A). The second component discriminate the non F-element chromosomes into two groups: one containing *Dp *chromosomes and the other containing the *Dm*/*Dy *chromosomes (Figure 1A). The same pattern was observed when the di-, tri-, tetra-and penta-mers were analysed (data not shown). However, a large amount of the variation in the first component can be explained by differences in nucleotide composition between the chromosomes (Figure 1B, Table 1). The sequence motifs that most strongly distinguish the F-elements contain only A/T nucleotides and are not very complex. To determine if more complex motifs can be used to separate the chromosomes, we need to remove most of the variation caused by the inequalities in their A/T contents. This was accomplished by dividing all scores by the expected scores, based on the chromosomal base composition. We then normalised the scores for all di-, tri-, tetra-, penta-and hexa-mer sequence motifs. After this normalisation the chromosomal separation was almost identical to the separation seen in the non-normalised PCA (Figure 1C shows results from the tetramer analysis) except when using penta-and hexamers.

**Table 1:** The length, number of N and A/T content of all chromosomes used in this study.

	Original sequence length	% N	% A/T	% removed by Tandem Repeats Finder	% A/T after Tandem Repeats Finder masking	% Removed by RepeatMasker	% A/T after RepeatMasker masking
*Dm*							
X	21780003	0.10	57.42	2.12	57.34	8.80	56.55
2L	22217931	0.01	58.08	0.81	58.07	6.59	57.54
2R	20302755	0.02	56.55	0.91	56.54	7.88	56.05
3L	23352213	0.05	57.92	0.87	57.93	6.77	57.36
3R	27890790	0.00	57.08	0.68	57.07	5.33	56.60
4(F)	1237870	0.08	64.71	1.17	64.53	26.70	64.58

*Dy*							
X	21591847	3.28	56.73	3.45	56.58		
2L	22678881	1.31	57.19	1.56	57.18		
2R	21288905	1.39	56.74	1.33	56.75		
3L	24977971	2.19	57.57	1.57	57.57		
3R	29717196	1.88	56.81	1.47	56.82		
4(F)	1395135	2.16	64.53	3.38	64.51		

*Dp*							
XL	24630256	4.10	54.22	2.86	54.33		
4	26108043	3.64	55.99	2.31	56.02		
3	19738113	3.41	53.52	1.50	53.61		
XR	24186629	3.87	53.76	3.63	53.96		
2	25998849	3.13	55.10	1.86	55.15		
5(F)	849497	25.81	61.45	1.02	61.42		

The percentages of base pairs removed by Tandem Repeats Finder and RepeatMasker as well as the A/T content of the remaining sequences are also given. The *Dp *chromosomes are listed in the same order as the corresponding *Dm *chromosomes.

**Figure 2:**
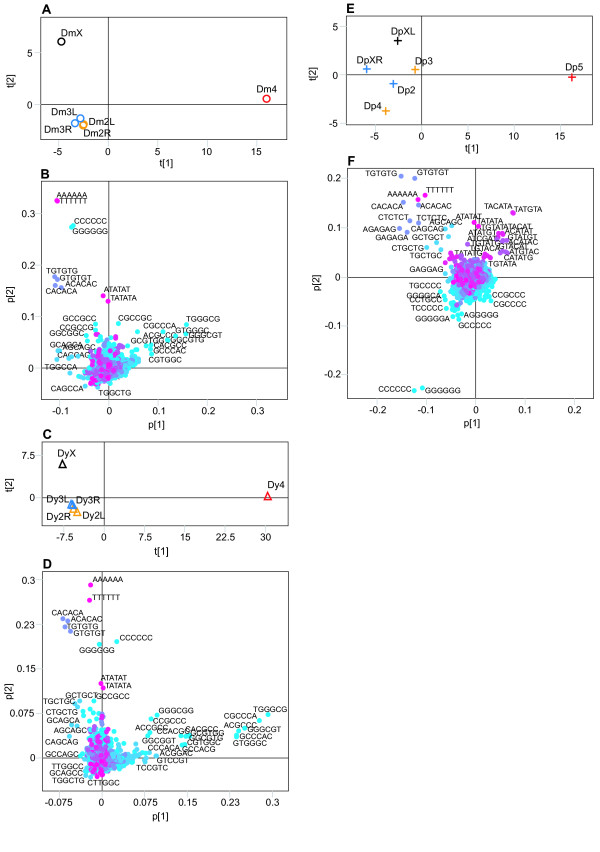
Results of the separate, normalised, whole chromosome PCA of the three genomes using hexamers. Chromosomes are colour-coded, as follows (according to the *Dm *numbering: black = X, yellow = 2, blue = 3 and red = 4). L and R stand for the left and right arms of the metacentric chromosomes, respectively. (**A**) Score plot (R2 = 0.97) of the *Dm *analysis. (**B**) Loading plot of the analysis in (**A**). (**C**) Score plot (R2 = 0.99) of the *Dy *analysis. (**D**) Loading plot of the analysis in (**C**). (**E**) Score plot (R2 = 0.92) of the *Dp *analysis. (**F**) Loading plot of the analysis in (**E**). The colouring of the hexamers in (**B**), (**D**) and (**F**) is proportional to the A/T content. Pink is all A/T and blue is all G/C.

**Figure 3:**
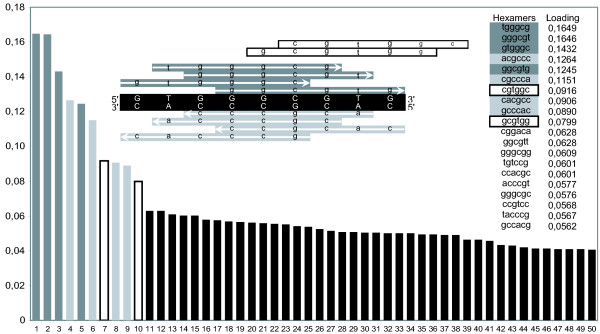
Graph showing the 50 hexamers with the highest loadings in the normalised *Dm *PCA. The combination of the eight hexamers into the nonamer is shown. The two hexamers not included in the motif are indicated by open boxes.

Analysis of the sequence motifs shows that the F-element separation is no longer solely explained by A/T motifs (Figure 1D). In the analyses using penta-and hexa-mer motifs the *Dp *F-element is more similar to the non F-element chromosomes and the *Dm*/*Dy *F-elements separates more from each other (data not shown). The reason for this became clear when the different genomes were separately analysed. In all three species, the F-element separated from the other chromosomes along the first component, regardless of the motif length used (results of the hexamer analysis are shown in Figure 2). In *Dm*/*Dy *the X chromosome was separated from the other chromosomes by the second component, although less markedly than the F-element. Interestingly, the left arm of chromosome X in *Dp *separates in the second component while the right arm clusters closer to the other chromosomes. This is in agreement with the hypothesis that the right arm of *Dp *X is a later addition [15]. The left arms of *Dm *X, *Dy *X and the *Dp *X are separated by the same hexamers. Many of the motifs causing the strong separation of the F-elements are the same in all three species. The top scoring penta-and hexamers can easily be aligned into longer motifs (Figure 3 shows results from the *Dm *hexamer analysis), all of which are supported by hexamers in both sense and anti-sense orientation.

For further analysis, we excluded parts of the longer motifs supported by only sense or only anti-sense hexamers. It should be noted that after the hexamers included in the longer motifs, there was a clear drop in loading (Figure 3). This suggests that a longer motif causes overrepresentation of the top scoring hexamers in the F-element. To verify the existence and overrepresentation of these predicted longer motifs, we counted their numbers on all chromosomes. The longer motifs are clearly more common on the F-elements compared to the other chromosomes (Table 2). In *Dm*/*Dy *the F-element motif is the same, but in *Dp *the motif is different (Table 2). We find the hexamers forming the longer motifs only in the normalised analysis. Our normalisation procedure assumes a random distribution of all nucleotides. If the nucleotide frequencies on, e.g. the *Dm *F-element would be highly influenced by micro satellites or more complex repeats, it would make the normalisation assumption invalid. However, this is not the case. When we removed all simple sequence repeats using Tandem Repeats Finder [17] or both simple and complex repeats using RepeatMasker [18] the chromosomal nucleotide compositions stayed roughly the same (Table 1).

**Table 2:** The number of longer motifs and pairs of motifs per Mbp on the different chromosomes.

	F-element motifs / Mbp	Pairs / Mbp	Sum of included hexamers / Mbp
*Dm*	gtgggcgtg/cacgcccac		
X	55.8	4.5	2069.1
2L	42.4	2.6	1770.3
2R	44.1	3.1	1926.8
3L	39.5	2.3	1761.3
3R	36.7	1.3	1867.7
4(F)	154.4	39.6	1875.7

*Dy*	gtgggcgtg/cacgcccac		
X	74.4	17.8	2247.3
2L	115.9	51.6	2319.5
2R	111.2	47.8	2337.3
3L	90.6	36.7	2132.7
3R	116.4	53.4	2399.1
4(F)	597.1	325.3	4810.2

*Dp*	tacatatgta		
XL	102.5	16.4	3269.7
4	44.8	4.1	2319.1
3	66.2	6.8	2588.3
XR	56.2	5.5	2408.1
2	41.5	3.7	2313.3
5(F)	244.3	23.8	6890.8

The sum of the hexamers making up the longer motifs per Mbp are also given.

Since *Dm *is by far the most intensively studied of the three species we concentrated our efforts on the nine base pair long motif found in the *Dm *F-element. Strikingly, in the F-element this nonamer is often found in pairs, i.e. two sense or two anti-sense nonamers are often situated close to each other. Furthermore, when we plotted the distances between the 192 motifs in the F-element we found that three different distances between them are overrepresented. Considering the 192 nonamers as 91 pairs, nine are separated by 17 (± 2) bp, 13 by 28 (± 1) bp and 10 by 79 (± 3) bp. In the subsequent analyses, we defined a pair as two nonamers separated by no more than 146 bp. According to this definition, 51% of the nonamers are organised in such pairs. The remaining nonamers seem to be randomly distributed in relation to each other. Only one pair on the entire F-element consists of one sense and one antisense nonamer. The nonamer pairs are even more enriched on the F-element than the nonamer (Table 2). To assess whether this frequency of pairs is higher than expected by mere chance we randomised the positions of all 192 nonamers in a simulation repeated 10 million times, and calculated the number of pairs in each case. Since less than 51% of the nonamers were paired in every run, we conclude that the observed nonamer pair frequency significantly exceeds the expected frequency. This strongly suggests that the nonamers exist in pairs, are important for the separation of the F-element in *Dm *and might confer a selective advantage. The *Dy *nonamers and *Dp *decamers also occur in pairs (Table 2).

**Figure 4:**
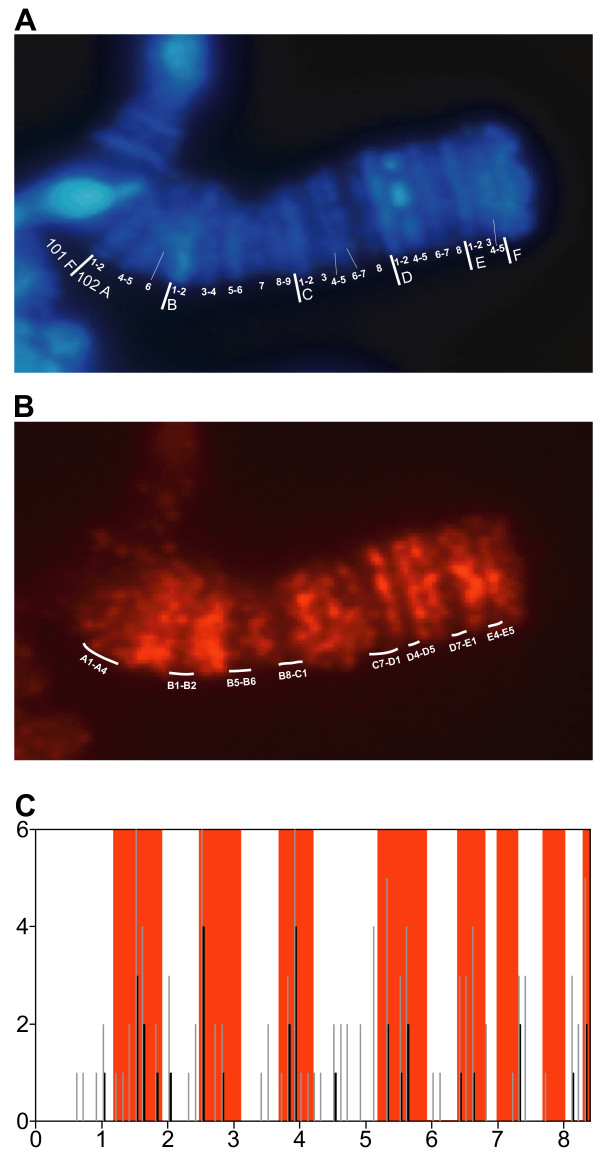
Localisation and mapping of POF on salivary gland F-element in *Dm*. (**A**) F-element stained with DAPI, showing cytological map positions. (**B**) F-element stained with anti-POF antibody. White lines indicate regions with weak POF staining. (**C**) Number of nonamers (grey) and nonamer pairs (black) per 10 kbp. The x-axis scale is in 100 kbp. White areas indicate regions of weak POF staining seen in (**B**). The sequence positions of the polytene chromosome bands are according to *Dm *annotation release 3.2. The genomic sequence 1 to 830 kpb shown in (**C**) corresponds to cytological region 102A to 102E5.

One of the atypical features of the *Dm *F-element is the specific binding of the protein POF. To determine if the nonamers or nonamer pairs are correlated to the binding of POF to the F-element, we mapped POF binding sites on polytene chromosomes (Figure 4A,B). It is difficult to map polytene bands beyond cytological position 102E5 so we limited this analysis to the region 102A-102E5. Comparison of the sequence positions of the nonamer pairs (Figure 4C) with the staining pattern of POF protein on the polytene F-element (Figure 4B) showed that regions with few or no pairs correlate well with regions lacking POF binding. The genomic sequence corresponding to the cytological regions that do not bind POF comprises 59% of the sequence from positions 1 to 830,000. 79% of the nonamer pairs and 61% of the nonamers are located outside these regions. We tested the significance of these results in a simulation, repeated 10 million times, in which we randomised the positions of the nonamers and the nonamer pairs. In all of these simulations the number of nonamers or pairs was lower than the observed numbers in the POF-binding regions.

The separation of the X chromosome seen in Figure 2A,C,E is due to simple sequences such as A_n_, T_n_, C/A_n _and G/T_n _repeats in both the non-normalised and the normalised analysis. This finding is in agreement with *in situ *hybridization data showing that C/A_n _and G/T_n _repeats are common on the X chromosome [19]. Positions of the hexamers that separate chromosome X show no clear correlation to the binding sites of the MSL complex defined by Demakova et al. [20] (data not shown).

**Figure 5:**
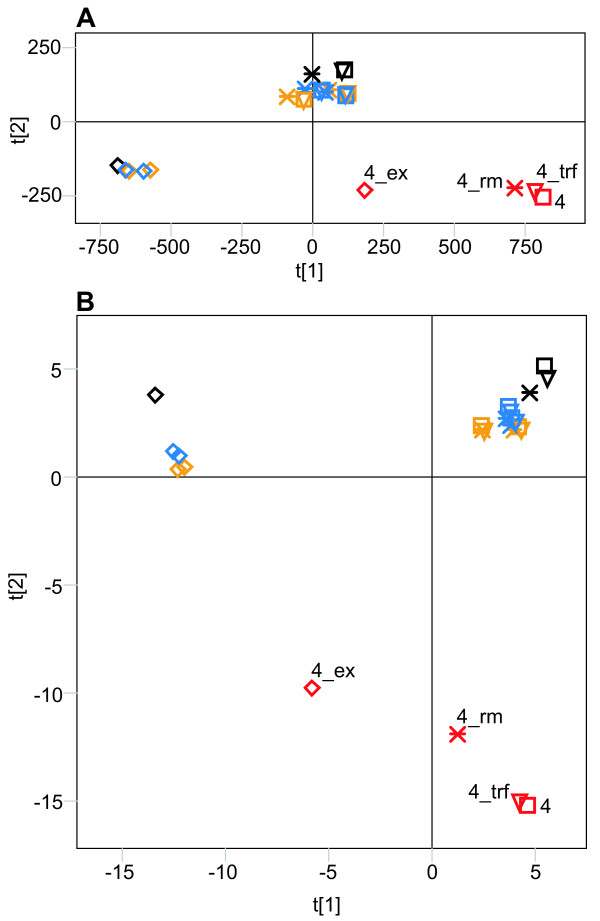
The combined PCA of four *Dm *data sets using hexamers. The four data sets used are: the original sequence (□), the Tandem Repeats Finder masked sequence (▽), the RepeatMasker masked sequence (*) and the extracted exon sequences (◇). (**A**) Score plot (R2 = 0.93) of the non-normalised analysis. (**B**) Score plot (R2 = 0.82) of the normalised analysis.

In an effort to determine the origin of the sequences causing the chromosomal separation in *Dm *seen in both the non-normalised and normalised PCA we repeated the analysis on three additional data sets. To evaluate the contribution of simple sequence repeats we masked the genome using Tandem Repeats Finder [17] and to evaluate the contribution of both simple and more complex repeats we used RepeatMasker [18]. We also merged all exon sequences of the different chromosomes. We then analysed the four datasets simultaneously, both with and without normalisation (Figure 5). The resulting plots show that the enrichment of simple A/T rich sequences on the F-element (seen in the non-normalised PCA, Figure 1B) cannot be explained by differences in repetitive elements. These sequence signatures were not removed by masking simple or more complex repetitive elements, implying that they are present in all non-exon sequences on the F-element (Figure 5A). Interestingly, the F-element exons do not share these sequences, but they still clearly separate from the exon sequences of the other chromosomes. Furthermore, the simple sequences that separate the X chromosome from the others distribute all over the non-exon sequences. In the PCA in which we accounted for differences in nucleotide composition, the separation was similar compared to the non-normalised analysis, except that the exons of the X chromosome separated from the exons of the other chromosomes (Figure 5B). It should be noted that the first component distinguishes between the exon sequences and the other sequences. The second component, however, separates all types of F-element sequences from the other chromosomal sequences. We conclude that the overrepresentation of some sequence signatures on the F-element cannot be attributed to either the high A/T content or the enrichment of repeated elements and that they are present in both exon and non-exon sequences. The general patterns we see are clearly not dependent on the type of sequence studied or differences in base composition.

In addition, we note that in the normalised PCA the RepeatMasked F-element separates more clearly from the original F-element sequence (Figure 5B) than in the non-normalised analysis. Many sequence signatures are shared by the F-elements in all four datasets. Examination of the top-scoring sequence motifs clearly shows that the RepeatMasked F-element lacks the nonamer motif described above (data not shown). We therefore studied the output file from RepeatMasker in further detail. According to RepeatMasker, 95.3% of the nonamer motifs reside within DINE-1 elements, and thus seem to be closely linked to them. The DINE-1 element has previously been shown by *in situ *hybridisation to be enriched on the *Dm *F-element [21]. We also note that in the DINE-1 sequence defined in the Repbase Update [22,23] there is a duplication of approximately 60 base pairs, each of which contains a nonamer pair, and in both pairs the individual nonamers are separated by 29 base pairs.

We also masked the genomes of *Dy *and *Dp *using Tandem Repeats Finder. Since these genomes have not yet been annotated we could not use exons or RepeatMasker. The PCA results of the original sequences and the masked sequences in these species are virtually identical (data not shown).

### Fragment analysis

In the whole chromosome analysis we identified sequence signatures that are enriched on different chromosomes, but we did not investigate their linear organisation along the chromosomes. Therefore, to find sequence signatures evenly distributed over the chromosomes that are capable of distinguishing one chromosome from the others, we fragmented each of the *Dm*, *Dy *and *Dp *genomes into 100 kb fragments. We then scored the positions of all possible di-, tri-, tetra-, penta-and hexa-mers in the 100 kb fragments of all chromosomes from each of the genomes. The first component of a PCA of these data mainly reflects differences in nucleotide composition between the fragments. Since the nucleotide composition can vary both between chromosomes and within single chromosomes we need to remove this variation in the dataset. One possibility would be to exclude the first component, but some of the variation caused by A/T skewing could still remain in the higher order components. To specifically remove the influence of variations in the base composition we created a Partial Least Squares (PLS) model using the non-normalised hexamer scores and the A/T content as a single response. We then used the residual matrix, after removing the variance described by the first component, for subsequent PCA analysis. The residual matrix is a normalised scoring matrix in which the variance in the data related to the base composition of the target sequence has been removed. The performance of the normalisation was evaluated by plotting the score values of the first component against the base composition of the fragments. As expected, the scores showed an almost perfect correlation with the base composition of the fragments (data not shown).

**Figure 6:**
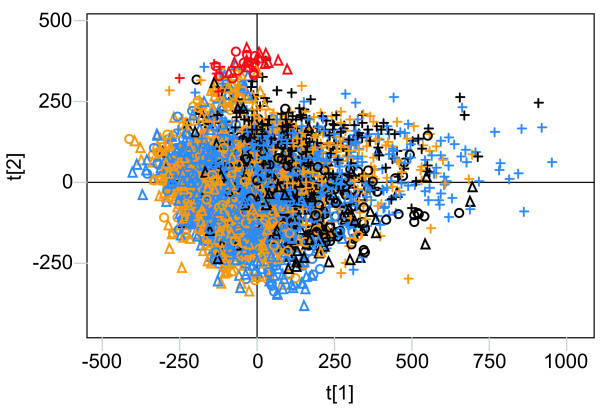
The hexamer PCA (R2 = 0.22) of 100 kb fragments (n = 3564) of the three genomes *Dm *(○), *Dy *(△) and *Dp *(+). Chromosomes are colour-coded, as follows (according to the *Dm *numbering: black = X, yellow = 2, blue = 3 and red = 4). The loadings are presented in Additional file 1.

PCA of the approximately 3600 fragments from all three species showed that the 33 F-element fragments cluster, and separate with minor overlaps from the other chromosomal fragments in the second component (Figure 6 shows results from the hexamer analysis). In the tri-and tetra-mer analyses, the overlap with other chromosomes was more extensive than in the di-, penta-and hexa-mer analyses (data not shown). In the first component of the hexamer PCA, roughly a third of the *Dp *fragments cluster separately from other chromosomal fragments. The third component separates many of the *Dm*/*Dy *X chromosomal fragments from the others, but only when using penta-and hexa-mers (data not shown). The sequence signatures responsible for the separation of the F-element are not the same as in the whole chromosome analysis and cannot easily be combined into longer motifs. For a full listing of the loadings for all 4096 hexamers for the first two components in the PCA see Additional file 1. In conclusion, the fragment analysis showed the existence of F-element-specific sequences that not only have been conserved for approximately 54.9 Myr, but also are linearly distributed along the sequenced part of the F-elements in *Dm*, *Dy *and *Dp*. Based on this conservation we speculate that there are sequence signatures that have a function for F-element identity.

**Figure 7:**
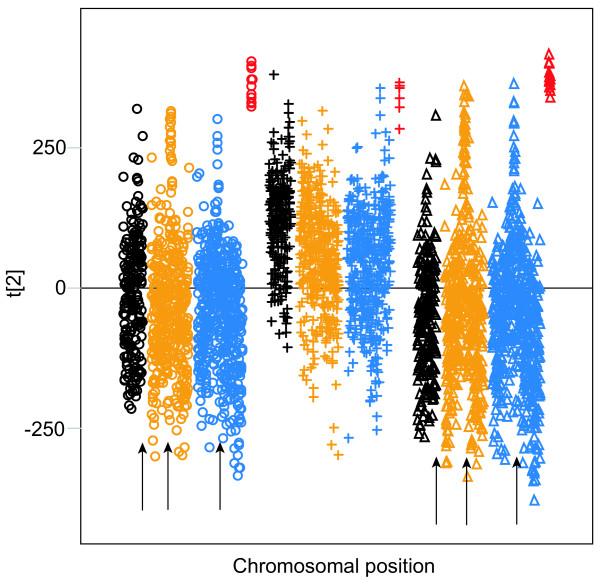
Scores from the second component in Figure 6 plotted against the linear order of the 100 kb fragments on the individual chromosomes from *Dm *(○), *Dy *(△) and *Dp *(+). Chromosomes are colour-coded according to the *Dm*/*Dy *numbering (black = X, yellow = 2, blue = 3 and red = 4). This should be noted when examining the *Dp *fragments. Proximal regions of *Dm *and *Dy *chromosomes are indicated by arrows.

When we plotted the scores from the second component (which separates the F-elements) against the chromosomal position we find that on average the *Dp *fragments are shifted towards the F-element fragments (Figure 7 shows results from the hexamer analysis). The centromere proximal regions of the non F-element chromosomes in all species are shifted towards the F-element fragments and the distal regions in the opposite direction. This pattern is not as clear in *Dp *as in *Dm *and *Dy*.

**Figure 8:**
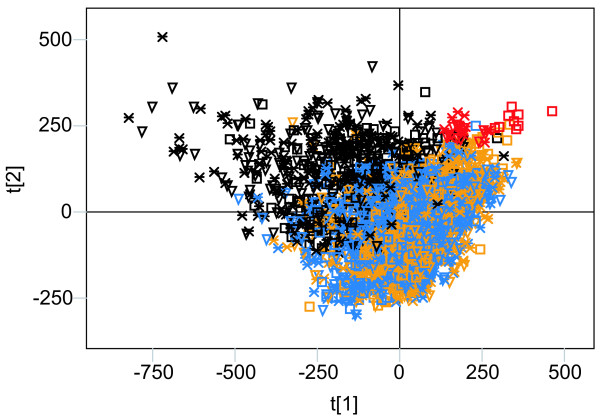
The combined PCA of 100 kb fragments (n = 3399) of three *Dm *data sets based on hexamers. The three data sets used are: the original sequence (□), the Tandem Repeats Finder masked sequence (▽) and the RepeatMasker masked sequence (*). Chromosomal origins of the fragments are indicated by colour (black = X, yellow = 2, blue = 3 and red = 4).

In the same way as for the whole chromosome study, we repeated the fragment analysis on chromosomes from the three species after masking them by Tandem Repeats Finder. The results from this masked dataset did not differ in any significant way from the prior analysis (data not shown). For *Dm*, we also masked the fragmented genome using RepeatMasker. A combined PCA with the original data, Tandem Repeats Finder masked data and RepeatMasker masked data showed that the F-element signatures distributed over the entire chromosome are not connected to either simple or complex sequence repeats (Figure 8 shows results from the hexamer results). In this analysis many X chromosomal fragments separated from the other fragments.

Interestingly, we note that in every PCA we performed most motifs had almost identical loading to their reverse complements. This was true for both the whole chromosome analysis and the fragment analysis, regardless of whether normalisation was applied and the motif length used. Baisnée et al. [24] have studied the reverse complement symmetry of DNA more thoroughly, but even though it seems to be universal, the underlying cause is not yet fully understood.

## Discussion

### Sequence signature analysis

In this work, we separately counted all di-(16), tri-(64), tetra-(256), penta-(1024) and hexa-mers (4096) and studied their distribution in the chromosomes of three *Drosophila *genomes using PCA. Short motifs (up to tetramers) can be rapidly scored and analysed. However, the frequencies of such short motifs are strongly influenced by the abundance of simple sequence repeats. Motifs longer than tetramers are less affected by simple sequence repeats, but are computationally more demanding to analyse. Sometimes, when a group of sufficiently long sequences, e.g. hexamers, are found to be overrepresented in a genomic sequence, they overlap and form longer sequences with higher discriminative power, thus increasing the chance of identifying longer and more complex sequences than if shorter sequences, e.g. trimers, are used.

The frequency of a sequence motif depends on both biological and stochastic factors. The expected frequency of a specific motif depends on the base composition of the chromosome. If the four nucleotides do not have equal frequencies in all chromosomes, the results from a non-normalised analysis will reflect the effects of a mixture of biological and stochastic factors. It is often difficult to isolate the effects of such factors, but a large part of the stochastic component can be removed by dividing all motif frequencies by the expected frequencies in a normalisation step. Otherwise, biologically interesting motifs may be masked by motifs that are common solely by chance. In this study, we used relatively basic normalisation procedures to account for differences in base composition. However, our multivariate approach could easily be extended to account for differences related to sequence complexity [see e.g. [25]] or any kind of prior knowledge about the target sequence.

### Whole chromosome analysis

In many respects, the F-element in *Dm *(the 4^th ^chromosome) is an atypical chromosome. It has an overall length of ~5 Mb, 3–4 Mb of which consists of simple satellite repeats and does not contain any known genes [26]. The remaining portion (1.23 Mb) has been sequenced and covers the cytogenetic bands 101E-102F on polytene salivary gland chromosomes. However, the banded portion appears to be a mosaic of unique DNA interspersed with moderate and low copy repetitive DNA [21,27-30]. The F-element is largely heterochromatic in nature. The heterochromatic protein HP1 and the modified histone, methylated H3Lys9, have been found to be associated with most of the F-element [31,32]. In accordance with its heterochromatic nature, the F-element has a higher A/T content compared to the other chromosomes. A high density of transposable elements (approximately six times higher than in the other chromosomes) is found in the *Dm *F-element [33]. Another interesting feature of the F-element is that it is decorated by the chromosome-specific protein, POF (Painting of fourth), which specifically "paints" the entire chromosome [6]. The F-element is an atypical autosome and has been suggested to have a closer kinship with the X chromosome than with the other autosomes [16,34]. The F-element has been suggested, partly on the basis of studies of the distant relative *D. busckii*, to originate from the X chromosome [35,36]. The binding of POF to the F-element is reminiscent of the binding of the *Drosophila *dosage compensation complex to the male X chromosome, which mediates its hypertranscription [reviewed by [4,5]]. In *D. busckii*, POF binds to the male X, further supporting the suggested relationship between the X chromosome and the F-element [6].

All chromosomes differ to some extent in nucleotide frequencies, with the F-element being extreme in this respect, having a high A/T content in all three species studied. When the raw data was analysed the F-elements in all three species separated collectively from the other chromosomes (Figure 1), due to differences in their contents of simple sequences containing only A and T. In *Dm *we performed the analysis on four datasets, derived from the original sequence, and the sequences obtained after masking simple sequence repeats, both simple and more complex repeats and after removing everything except the exon sequences. The results show that the simple A/T sequences, which separate the F-element in the original data, are distributed throughout the non-exon F-element sequences and cannot be attributed to microsatellites and transposable elements. It should also be noted that the F-element exons separate equally well from the exons of other chromosomes. The X chromosome also separates from the other chromosomes, albeit to a lesser extent, due to differences in their simple sequences. The same chromosomal separation is seen regardless of the motif length used. As shown in Figure 1, all of the *Dp *chromosomes are shifted relative to the *Dm*/*Dy *chromosomes, suggesting the presence of *Dp*-specific signatures in addition to the chromosome-specific signatures studied here.

To detect more complex and potentially functional motifs hidden by the skewed base composition, we normalised our scores according to the base composition of each chromosome analysed. As shown in Figure 1C, the resulting separation was nearly identical to that seen in the non-normalised analysis (Figure 1A). The *Dm *F-element was clearly separated even after removal of repeated elements from the genome (Figure 5B). It should be noted that the first component in this PCA (Figure 5B) distinguishes the exons from non exon sequences. In the second component, however, all F-element sequences including the exon sequences, cluster together. We conclude that the F-element exons also contain F-element signatures.

The separate analysis of the three species showed that the pentamer and hexamer motifs that are most important for distinguishing the F-element can be aligned into longer sequences. Examination of the top scoring hexamers clearly shows that they are part of a nonamer in *Dm *and *Dy*, and of a decamer in *Dp*. These sequences are strongly enriched in the respective F-elements (Table 2), although the individual hexamers in *Dm *are not enriched in the non-normalised analysis. Since *Dm *is the only annotated species, we concentrated our investigation on the *Dm*/*Dy *nonamers. Plotting the positions of these nonamers in the *Dm *F-element showed that they commonly occur in pairs, separated by no more than 146 bp, all but one of which consists of two sense or two anti sense nonamers. The individual nonamers are enriched roughly four-fold in the F-element, while the pairs are enriched about 15-fold. The nonamers and decamers are also organised in pairs in *Dy *and *Dp *respectively (Table 2). We conclude that even though the method is based on relatively short sequence motifs, it still provides a potent means for finding longer and more complex sequence motifs.

Since POF is a protein that specifically paints the *Dm *F-element, we tested the possibility that the nonamer or nonamer pairs may be correlated to POF-binding sites. For this purpose, we stained polytene chromosome preparations using POF antibodies. After carefully mapping the banded regions, we compared the positions of nonamer pairs to the POF staining pattern. The genomic regions with few or no pairs correlate well with regions on the F-element that do not bind POF (Figure 3). We hypothesise that the nonamer pairs have a function and are directly or indirectly involved in POF binding to the F-element in *Dm*. However, this hypothesis needs to be verified experimentally. Since POF will not bind to a translocated *Dm *F-element [6] the nonamer pairs are not sufficient by themselves for recruiting POF. If the pairs have a function, it is possible that some variation is allowed within the nonamer and that there are motifs of differing strength. According to our RepeatMasker analysis of the F-element, 95.3% of the nonamers are located within DINE-1 elements. As shown in Figure 2B, the hexamers forming the nonamer are important for the separation of the F-element. Nevertheless, after removing virtually all of the nonamers using RepeatMasker (Figure 5B) F-element separation was retained, indicating that other signatures, apart from the nonamer, help distinguish the *Dm *F-element.

In an extensive study of local deletions flanking a transpsoson reporter Sun et al. [37] showed that the genomic region 400000 to 440000 of the *Dm *F-element is euchromatic. A nearby region induces gene silencing and is therefore considered to be heterochromatic. Sun et al. [37] attribute this to local induction of heterochromatin by the 1360 (hoppel) element. According to their model the 1360-induced heterochromatin can spread, but only ~10 kb or until encountering competition from euchromatic determinants. In this context, it should be stressed that this "euchromatic" region is enriched in DINE-1 fragments containing the nonamer pair region. We speculate that these nonamer-containing DINE-1 fragments act as euchromatic determinants. We have previously proposed that POF is involved in a chromosome-specific gene regulatory mechanism [7]. It should be noted that according to our cytological mapping POF binds within this euchromatic region (370000 to 430000).

### Fragment analysis

In the whole chromosome analysis we identified sequence signatures that are overrepresented in different chromosomes, but we did not study the linear organisation of the sequence signatures along the chromosomes. Instead, we divided each of the *Dm*, *Dy *and *Dp *chromosomes into 100 kb fragments to check for the presence of sequence signatures that can distinguish fragments of specific chromosomes from those of other chromosomes, especially signatures distributed over the whole chromosome. For such an analysis it is important to remove all variation connected to differences in nucleotide composition. Using a Partial Least Squares (PLS) model with A/T composition of every fragment as a single response we removed this bias. Strikingly, when the approximately 3600 fragments from all three species were analysed using PCA based on di-, penta-and hexa-mers the 33 F-element fragments clustered together (Figure 6). The motifs responsible for this separation were not the same as in the whole chromosome analysis. Nevertheless, this demonstrates the existence of sequence signatures that are capable of separating all F-element fragments from the three different species. Based on the relationship of these species we conclude that these signatures have been conserved for at least 54.9 Myr [15]. These conserved motifs are also linearly distributed along the sequenced part of the F-elements (Figure 6). The F-elements from the three species have high A/T contents and are probably all enriched in mobile and repeated elements. However, the motifs separating the F-element fragments are not connected to simple sequence repeats since masking such repeats did not alter the results. In addition, the *Dm *F-element fragments clustered together when the original sequence was analysed together with sequences in which both simple and complex repeated elements had been masked (Figure 8). Therefore, the collective separation of F-element fragments in the three species cannot be attributed to any known repeated elements, and we speculate that the signatures we identified have a role in F-element identification. The X chromosomal fragments of *Dm*/*Dy*, but not *Dp*, can also be separated to some degree using penta-and hexa-mers.

As shown in Figure 7, some non F-element fragments are more similar to the F-element fragments. These non F-element fragments are the centromere proximal regions of *Dm*/*Dy *chromosomes 2 and 3. The heterochromatic nature of the F-element in *Dm *is well established, e.g. by its enrichment of HP1 and H3K9 methylation [31]. In our analysis, the proximal regions of chromosomes 2 and 3 in *Dm*/*Dy *showed similarity to the F-element. It is interesting that an anti-metH3K9 antibody decorates the proximal regions of chromosomes 2 and 3 as well as the F-element in *Dm*. The proximal region of X is also stained, but to a much lesser extent using this antibody (JL unpublished results). We note that the same pattern is present in Figure 7. We must consider the possibility that chromatin similarities cause the partial overlap of the F-element and the proximal regions of chromosomes 2 and 3 (and that the heterochromatic nature of the F-element caused its observed separation from the other chromosomes). It is difficult to fully separate chromosome-and chromatin-specific effects. Sequences that have high A/T contents and are enriched in repetitive elements tend to be heterochromatic. As shown in Figures 5 and 8, the F-element separation was retained after normalising for differences in A/T content. Furthermore, the results were not significantly different when simple sequence repeats were removed using Tandem Repeats Finder, or when simple sequence repeats and repetitive elements were removed using RepeatMasker. The findings even apply to the exon sequences. Thus, we conclude that our methodology is capable of detecting chromosome-specific sequences.

## Conclusion

We have shown that the F-elements of three species that separated roughly 55 Myr ago share sequences that are distributed over the entire chromosomes. These sequences are not related to their unusually high A/T contents or any known repeated elements. In conclusion, our results support the existence of sequence signatures that confer chromosome specific integrity in *Drosophila*.

## Methods

### Hexamer scoring

We scored all positions of all possible di-(16), tri-(64), tetra-(256), penta-(1024) and hexamers (4096) in the genome sequence of *Dm*, *Dy *and *Dp*. Every motif was counted in each target sequence. Full-length chromosomes and 100 kb fragments were used as targets. Scoring was done by a sliding window approach, sliding one nucleotide at a time. The scoring function gives a two dimensional data-matrix with target sequences as objects (rows) and the total score for each motif as variables (columns). By dividing each element in the matrix by the length of its target sequence a relative score is obtained. Prior to analysis all data were mean-centred, i.e. each value was adjusted by subtracting the average value for the corresponding variable. All scoring and data normalisation procedures were performed using custom software developed in C, Java and Perl. The software can be obtained, on request, from the corresponding author.

### Multivariate analysis

#### Principal Component Analysis – PCA

The central idea of PCA is to extract a few, so-called, principal components describing most of the variation present in the data. The principal components are linear combinations of the original variables and uncorrelated to each other.



where *t *are the scores, *p *the loadings, *A *is the number of principal components and *E *is the residual matrix. The principal components can be determined using the NIPALS algorithm [38] or by Singular Value Decomposition (SVD) [39]. The scores (*t*) show how the objects and experiments relate to each other. The loadings (*p*) reveal variables that have an important influence on the patterns seen in the score plot.

#### Partial Least Squares – PLS

PLS is a multivariate regression method that relates the data matrix (*X*, the scoring data) to single (*y*) or multiple (*Y*) response(s). PLS has proved to be a powerful tool for finding relationships between descriptor matrices and responses, especially when there are more variables than observations and the variables are co-linear to each other and noisy. In our study, PLS was used to normalise the data by removing the variance in the scoring data that was correlated to the A/T content of the chromosome fragments. The PLS theory and methods discussed here concern single *y*-responses. As in PCA, principal components are constructed to reduce the dimensions of *X*. In order to obtain the principal components, PLS maximizes the covariance between the response variable *y *and a linear combination of the original variables *t *= *Xw*, where *t *is the score vector, *X *is the data matrix and *w *is the weight vector. For a more in-depth description of PLS, see [40-42] and references therein.



where *t *is the score vector for *X*, *A *is the number of PLS components, *p *is the loading vector for *X*, *c *is the loading vector for *Y*, *E *is the residual matrix for *X *and *F *is the residual matrix for *Y*.

All multivariate analyses and visualisations were performed using the Evince software package .

### Data normalisation

#### Probability normalisation

The probability of successfully aligning a motif to a target depends on the base composition of the motif sequence and the target sequence. For example, the chance of finding a given A/T-rich motif is relatively high in an A/T-rich target due to their similarity in base composition. Probability normalisation removes this systematic bias from the data. Each value is normalised by dividing the observed number of hits by the expected number of hits. The initial scoring is performed as described above, except that the scores are not divided by the target sequence length. The number of expected hits was calculated as follows:



where *N *is the target sequence length, *i *= {G,A,T,C}, *f*(*i*) = frequency of base *i *in the target sequence and *n*_*i *_= count of base *i *in the hexamer.

#### Fragment normalisation

To remove all variance in the scoring matrix obtained from the 100 kb fragment analysis that was solely related to the base composition of the target sequences, a different normalisation was applied, in which we created a PLS model with the base composition of every fragment as a single *y*-response and the scoring matrix as an *x*-matrix. By removing the variance explained by the first component a residual matrix was obtained, in which all variation caused by differences in base composition amongst the fragments had been removed. The residual matrix *E *was calculated as follows:

*E *= *x - tp*'

Where *x *is the hexamer scoring matrix, *t *= PLS-scores for the 1^st ^component and *p' *= PLS-loadings for the 1^st ^component.

The normalised data were then used for PCA analysis of the fragmented genome.

### Repeat masking

RepeatMasker [18] was run using default parameters, MaskerAid [43] and the *Drosophila *library file from Repbase [22,23]. Tandem Repeats Finder [17] was run using default parameters and a maximum period size of 500.

### Polytene chromosome staining

Polytene chromosomes from 3^rd ^instar larvae of wild type *Dm *were prepared and stained essentially as previously described [44]. Salivary glands were fixed in 2% formaldehyde in PBS, 0.1% Triton X-100, 0.2% NP-40 for 30 seconds followed by 2 minutes in 50% acetic acid, 1% formaldehyde. Polytene chromosomes were squashed as previously described [44]. The slides were washed for 30 minutes in 1 × PBS, 0.1% Triton X-100, transferred to blocking solution (0.1 M maleic acid, 0.15 M NaCl, 1% Boehringer blocking reagent) and incubated for 30 minutes at room temperature. The slides were then incubated overnight at 4°C with a rabbit polyclonal anti-POF primary antibody [6]. The slides were washed for 2 × 10 minutes in 0.1 M maleic acid, 0.15 M NaCl, 0.3% Tween 20 and blocked for 30 minutes. As a secondary antibody, a donkey anti-rabbit conjugated with Cy3 (Jackson Laboratories) was used, diluted 1:400 and incubated at room temperature for 2 hours. The squashes were counterstained with DAPI (1 μg/ml) and washed for 2 × 10 minutes before mounting with Vectashield (Vector). Chromosomes were analysed using a Zeiss Axiophot microscope equipped with a KAPPA DX20HC CCD camera. Images were assembled, contrasted and merged electronically using Adobe Photoshop. Well spread F-elements were mapped according to Saura et al. [45] and POF-binding regions were defined. To correlate cytological positions to sequences we used the *Dm *genome release 3.2. Since all sequences are annotated to cytological bands and POF binds preferentially to interbands we used regions with no POF binding for comparison. This is the reason why regions lacking POF binding are used for the correlation study.

## Authors' contributions

All authors were involved in the initial project discussion. PS and FP did the detailed planning, carried out all computational work, performed the analysis and wrote the draft manuscript. JL carried out the chromosome staining and participated in the final analysis. AOS carried out the mapping. All authors contributed to, read and approved the final manuscript.

## Supplementary Material

Additional File 1 The loadings of the PCA in Figure 6. Click here for file
